# Rhizosphere Engineering by Root Exudates: High‐Yielding Alfalfa Recruits Functional PGPR to Sustain Soil Nutrient Availability Under Long‐Term Cultivation

**DOI:** 10.1111/pce.70631

**Published:** 2026-07-29

**Authors:** Yanliang Sun, Kongqin Wei, Kaixin Yang, Jinfeng Hui, Dongjie Xia, Xuzhe Wang, Andrew D. Cartmill, Ignacio F. López, Chunhui Ma, Qianbing Zhang

**Affiliations:** ^1^ College of Animal Science and Technology, Bingtuan Key Laboratory for Efficient Utilization of Non‐Grain Feed Resources Shihezi University Shihezi China; ^2^ School of Agriculture and Environment Massey University Palmerston North Manawatū‐Whanganui New Zealand

**Keywords:** *Medicago sativa* L, rhizobox, rhizosphere interactions, root exudates, sustainable agriculture

## Abstract

Soil nutrient transformation capacity is a critical determinant of sustainable productivity in perennial cropping systems; however, the extent to which high‐yielding crops actively regulate rhizosphere microbial assembly to maintain nutrient availability remains poorly understood. We investigated whether root exudates from high‐yielding alfalfa (*Medicago sativa* L.) selectively recruit plant growth‐promoting rhizobacteria (PGPR) to enhance nutrient transformation. In an 8‐year continuous alfalfa system (2018–2025), high‐yielding cultivars increased soil organic carbon by 8.64%, total nitrogen by 6.01%, and moderately labile phosphorus fractions by 1.62%. Rhizobox experiments demonstrated that root exudates enhanced growth only with an active microbiome. High‐yielding alfalfa enriched PGPR communities, specifically *Ensifer*, *Pseudomonas*, and *Bacillus*. Isolated strains exhibited N fixation, P solubilisation, and IAA production. Metabolomic profiling revealed that exudates were enriched in specific sugars and amino acids. Maltopentaose, maltotetraose, taurine, N‐acetyl‐L‐leucine, and asparagine functioned as chemoattractants, stimulating PGPR proliferation and biofilm formation. These findings demonstrate that root exudate‐mediated, targeted recruitment of functional PGPR enhances N fixation and P transformation, thereby supporting sustained high alfalfa productivity. This study demonstrates a key rhizosphere mechanism underlying the long‐term sustainability of high‐yielding perennial legume systems and provides a mechanistic basis for microbiome‐informed sustainable alfalfa production and management.

## Summary statement

Although maintaining soil nutrient availability is critical for perennial productivity, how high‐yielding crops regulate rhizosphere microbial assembly remains poorly understood. This study demonstrates that high‐yielding alfalfa sustains long‐term productivity by secreting specific root exudate metabolites, such as maltopentaose and taurine, to selectively recruit functional PGPR (e.g., Ensifer, Pseudomonas) for enhanced nitrogen fixation and phosphorus transformation, revealing a key rhizosphere engineering mechanism for sustainable legume systems.

## 1 Introduction

Climate change and progressive degradation of arable land have been constraining the ability to sustain high productivity in forage systems for modern production agriculture (Chowdhuri and Pal 2025). Alfalfa (*Medicago sativa* L.), the most widely cultivated perennial forage legume, plays a pivotal role in livestock production and agroecosystem sustainability due to its high yield potential, superior forage quality, and ecological functions (Annicchiarico et al. 2014; He et al. 2025; Wei et al. 2025). At early flowering, alfalfa typically contains > 20% crude protein (CP) with high digestibility, underpinning its global importance as a source of high‐quality forage (Hadidi et al. 2023). As a deep‐rooted perennial capable of symbiotic biological N (N_2_) fixation via root nodules, alfalfa also contributes to soil structural improvement, nutrient accumulation, and long‐term soil fertility (Tian et al. 2018).

Despite these advantages, maintaining sustained high productivity in alfalfa systems remains challenging. While high initial soil fertility or intensive fertilisation can support short‐term yield gains (Xu et al. 2024), productivity often declines after several years of continuous cultivation. Many high‐yielding cultivars exhibit pronounced yield reductions after 3–5 years, a phenomenon increasingly described as “unsustainable high yield” (Wang et al. 2024a). This decline reduces land‐use efficiency and increases reliance on external nutrient inputs. Conventional explanations attribute yield loss to continuous cropping obstacles, pathogen accumulation, or progressive soil nutrient depletion (Liao et al. 2025). However, emerging evidence suggests that shifts in rhizosphere microbiome structure and function, particularly those affecting nutrient transformation processes, are critical determinants of long‐term productivity in perennial systems (Xueping et al. 2025).

Plants are not passive recipients of soil microbial processes but actively regulate their rhizosphere through the release of low‐molecular‐weight root exudates, a phenomenon often termed “rhizosphere regulation” (Wang et al. 2025a). Root exudates, including sugars, amino acids, organic acids, phenolics, and secondary metabolites, serve both as microbial C substrates and as signalling compounds that selectively shape microbial community assemblage. For example, maize (*Zea mays* L.) enhances organic acid exudation under P limitation to recruit P‐solubilising bacteria (Zhang et al. 2023), while taxifolin released by potato (*Solanum tuberosum* L.) roots functions as a chemoattractant for *Bacillus* spp. in potato‐tomato (*Solanum lycopersicum* L.) intercropping systems (Zhou et al. 2023). Similarly, oat (*Avena sativa* L.; Zhalnina et al. 2018) and cucumber (*Cucumis sativus* L.; Wen et al. 2020) dynamically adjust root exudate profiles under abiotic stress, assembling microbiomes that enhance stress resilience. Collectively, these studies demonstrate that plants can ‘engineer’ their rhizosphere microbiomes via root exudate‐mediated selection to enhance nutrient acquisition and their environmental resilience.

Within these microbiomes, plant growth‐promoting rhizobacteria (PGPR) represent a key functional guild associated with healthy and productive plant systems. Dominant PGPR taxa commonly include *Bacillus*, *Pseudomonas*, and *Arthrobacter* (Verbon and Liberman 2016; Saraf et al. 2014). These microorganisms promote plant growth through multiple mechanisms, including biological N_2_ fixation (Walker et al. 2010), P solubilisation (Liu et al. 2023), phytohormone production (Gowtham et al. 2022), mitigation of abiotic stress (Sharma et al. 2024), and suppression of soil‐borne pathogens (Oulad Ziane et al. 2024). Importantly, PGPR contribute directly to soil nutrient transformation by converting recalcitrant nutrient pools in plant‐available forms. For example, P‐solubilising PGPR release organic acids or phosphatases that mobilise stable P fractions (Liu et al. 2023), while interactions between PGPR and other N‐cycling microorganisms enhance rhizosphere N bioavailability (Walker et al. 2010). Thus, PGPR function as central mediators of nutrient exchange at the plant‐soil interface, linking microbial metabolism to sustained crop productivity.

Although modern breeding programs have historically focused on aboveground yield traits, increasing evidence indicates that sustained productivity depends critically on root and rhizosphere regulatory capacity (Vullaganti et al. 2025; Wang et al. 2025b). High‐yielding cultivars of several crops maintain efficient root exudation strategies that promote beneficial microbial recruitment and nutrient efficiency. For example, high‐yielding genotypes of barley *(Hordeum vulgare* L.; Zhu et al. 2023) and peanut (*Arachis hypogaea* L.; Cesari et al. 2019) secrete organic acids and flavonoids under nutrient stress to selectively recruit N‐fixing and P‐solubilising PGPR. These findings suggest that sustained productivity arises not solely from genetic yield potential or external inputs, but from coordinated plant‐microbe interactions mediated by root exudates. However, whether high‐yielding alfalfa cultivars similarly optimise exudate composition to recruit functional PGPR and enhance N and P bioavailability remains poorly understood, limiting the application of rhizosphere‐based strategies in sustainable forage systems.

Moreover, most existing studies are based on short‐term experiments or single growth stages, providing limited insights into rhizosphere processes operating over multi‐year cultivation cycles. Although long‐term high‐yielding alfalfa cultivation is known to alter soil nutrient pools, it remains unclear whether these changes are driven by cultivar‐specific root exudation patterns that selectively enrich nutrient‐transforming PGPR. In particular, the chemodiversity of alfalfa root exudates and the identity of key metabolites responsible for PGPR recruitment have not been systematically examined.

To address these gaps, we compared alfalfa cultivars with contrasting yield performance under long‐term continuous cultivation to investigate how root exudates regulate PGPR colonisation and soil nutrient transformation. We test three hypotheses: (i) High‐yielding alfalfa increases soil organic carbon (SOC) and alkaline hydrolyzable N (AHN), while promoting the conversion of stable P pools into labile forms through microbially mediated processes; (ii) High‐yielding cultivars selectively recruit PGPR with enhanced nutrient‐transforming functions relative to low‐yielding cultivars; and (iii) These microbial differences are driven by specific key differential metabolites in root exudates of high‐yielding alfalfa.

## 2 Materials and Methods

### 2.1 Field Study

The study was established on April 27, 2018, at the Grassland Experimental Station of Shihezi University, Xinjiang (44°20′ N, 86°30′ E, 420 m a.s.l.). The site is characterised by a temperate continental arid climate, with a mean annual temperature of 5°C–10°C, a frost‐free period of 167–172 days, and annual precipitation of 190–260 mm. The soil is classified as a grey desert soil (Chinese Soil Classification) with generally low inherent fertility.

A single‐factor randomised complete block design was employed, comprising 95 alfalfa cultivars, each with three replicates. Individual plots measured 30 m^2^ (3 m × 10 m) and were separated by a 1.5 m buffer zone between plots. Seeds were manually sown at a depth of 2 cm, with 20 cm between seeds on the row, and a seeding rate of 18 kg·ha^−1^, followed by light soil compaction to ensure good soil‐seed contact. A subsurface drip irrigation system was installed at 8–10 cm depth with a drip tape spacing of 60 cm, providing an annual irrigation volume of approximately 6750 m^3^·ha^−1^. Irrigation was applied before and after each harvest to maintain uniform soil moisture in the root zone. No chemical fertilisers were applied during the experimental period. Weed and pest control followed standard regional management practices for regional high‐yield alfalfa systems. Plants were manually harvested at the early flowering stage (5%–10% bloom), leaving a stubble height of 5 cm. Based on local climatic conditions and growth cycles, yields from three harvests were recorded in 2024 and 2025. For each plot, ten uniformly growing plants were randomly selected, harvested, cleaned of debris, and immediately weighed to determine individual aboveground fresh biomass.

### 2.2 Determination of Soil Physicochemical Properties

Soil sampling was collected during the early flowering stage on 12 August, 2025. Rhizosphere soil samples were taken on alfalfa rows at 5 cm from the base of individual plants, from the 0–100 cm soil profile at 10‐cm continuous depth intervals using a soil auger, with three replicates per plot. Electrical conductivity (EC) was measured in a 1:5 (soil:water) extract using a portable conductivity metre after shaking and centrifugation. Soil pH was determined in a 1:2.5 (soil:water) suspension using a pH. Soil OC was analysed via potassium dichromate oxidation with external heating (Bao 2000). Total N (TN) was determined by the Kjeldahl method (Bao 2000), while AHN was determined by alkaline diffusion (Bao 2000). Total P (TP) was measured following H_2_SO_4_─H_2_O_2_ digestion using molybdenum‐antimony resistance colourimetry (Bao 2000), while available (AP) was assessed using the Olsen method combined with molybdenum blue colourimetry (Bao 2000).

Soil P fractions were determined using a modified Hedley sequential extraction (Hedley et al. 1982; Guppy et al. 2000), yielding: H_2_O‐P, NaHCO_3_‐Pi, NaHCO_3_‐Po, NaOH‐Pi, NaOH‐Po, dilute HCl‐P, concentrated HCl‐Pi, concentrated HCl‐Po, and residual‐P. Based on biological availability, these fractions were categorised as labile P (H_2_O‐P, NaHCO_3_‐Pi, and NaHCO_3_‐Po), moderately labile P (NaOH‐Pi, NaOH‐Po, and dilute HCl‐P), and stable P (concentrated HCl‐Pi, concentrated HCl‐Po, and residual‐P).

### 2.3 Partitioned Cultivation Experiment

Partitioned cultivation experiments were conducted using plastic containers (length × width × depth: 48 cm × 18 cm × 12 cm) divided into four equal compartments. The central compartment was fully isolated, while the lateral compartments were separated by one of three barrier treatments: (i) Inter (no barrier, allowing full root contact and chemical exchange; (ii) REE (30 µm nylon mesh allowing root exudate diffusion but preventing root penetration); and (iii) Mono (a solid barrier preventing all physical and chemical interactions) (Zhou et al. 2023).

To evaluate microbial mediation, both sterilised and non‐sterilised soil treatments were included. Sterilised soil was autoclaved (121°C, 0.1 MPa, 60 min), while non‐sterilised soil retained its native microbial community. All substrates (sterilised and non‐sterilised soils) were mixed with perlite at a 3:1 (v/v) ratio and homogenised prior to filling of the compartments/containers. The soil was sourced from the Grassland Experimental Station of Shihezi University, Xinjiang, with basic physicochemical properties: pH 8.22, organic matter (OM) 25.12 g·kg^−1^, and AHN 38.23 mg·kg^−1^, AP 12.37 mg·kg^−1^.

Healthy alfalfa seeds were surface‐sterilised using 75% ethanol for 30 s, followed by immersion in 10% sodium hypochlorite for 5 min and thorough rinsing with sterile water to eliminate residual reagents. Seeds were placed in Petri‐dishes and pre‐germinated in the dark at 25°C for 12 h (until radicle emerged), and then sown at 1.5 cm depth.

A completely randomised design was adopted, with 12 independent plastic containers (pots) as replicates for each treatment. After emergence, seedlings were thinned to three uniform plants per container. Containers were maintained in a greenhouse at 25/20°C (day/night), with a 14 h photoperiod and 60%–70% relative humidity. Plant height and aboveground fresh biomass were measured at the early flowering stage of the first and second harvests.

### 2.4 DNA Extraction and High‐Throughput Sequencing

Rhizosphere soil samples were collected at the early flowering stage of the final alfalfa cut/harvest to analyse bacterial community structure. Loosely adhering soil was dislodged from the alfalfa roots by gentle shaking, while soil tightly bound to root surfaces was collected as rhizosphere soil. Samples were sieved (2 mm), homogenised, and stored at −80°C. Genomic DNA was extracted from 0.5 g of soil using the DNeasy PowerSoil Kit (Qiagen, Germany). DNA integrity was verified by agarose gel electrophoresis, and concentrations were quantified using a Nanodrop 2000 spectrophotometer. Bacterial 16S rRNA gene libraries targeting the V3–V4 region were amplified using primers 341 F (5′‐CCTACGGGNGGCAGCAG‐3′) and 806 R (5′‐GGACTACHVGGGTMTCTAAT‐3′) sequenced on an Illumina NovaSeq. 6000 platform by Shanghai Biochip Co. Ltd. PCR conditions consisted of an initial denaturation at 95°C for 5 min, followed by 25 cycles of 95°C for 30 s, 50°C for 30 s, and 72°C for 40 s, with a final extension at 72°C for 7 min. All 16S rRNA sequence data from the field soil of alfalfa have been deposited in the NCBI BioProject under the accession number PRJNA1416912.

### 2.5 Collection and Metabolomic Analysis of Root Exudates

Root exudates were collected using the soil‐cultivation leaching method. Rhizosphere soil was collected aseptically, pooled from at least three adjacent plants per replicate, sieved (20‐mesh screen; aperture ~0.85 mm), and transported on ice to the laboratory for immediate processing or storage at −80°C. Fresh soil (100 g) was leached with 500 ml deionized water at 4°C for 3 h with agitation. After centrifugation (8000 × *g*, 5 min), the supernatant was filtered (0.22 µm), concentrated under reduced pressure, dissolved in methanol, evaporated under N, and reconstituted for analysis (Pantigoso et al. 2020).

Non‐targeted metabolomic profiling was performed using ultra‐high‐performance liquid chromatography‐tandem high‐resolution mass spectrometry (UHPLC‐MS/MS). Chromatographic separation was achieved on a Vanquish system (Thermo Fisher, Dreieich, Germany) with a Hypersil Gold C18 column (100 × 2.1 mm, 1.9 μm), using methanol‐water as the mobile phase under gradient elution with positive/negative ion switching (Sellick et al. 2011). Mass spectrometry was conducted on an Orbitrap Q Exactive™ HF platform (Thermo Fisher, Dreieich, Germany), providing high resolution and mass accuracy for precise metabolite detection. Differential metabolites were identified using the criteria of |log2(fold change) | > 1, and *p*‐value < 0.05.

### 2.6 Isolation and Plant Growth‐Promoting Trait Characterisation of Rhizobacteria

Healthy alfalfa plants were selected for rhizosphere soil collection. Rhizosphere soil suspensions were serially diluted and plated on LB agar. Distinct colonies were purified and preserved in 30% glycerol at −80°C. Phosphate solubilisation, N fixation potential, and indole‐3‐acetic acid production were assessed using standard plate and liquid assays (Zhang et al. 2024). Strains exhibiting multiple plant growth‐promoting traits were identified by 16S rRNA gene sequencing (Guan et al. 2023).

Preparation of Culture Media All culture media were prepared using distilled water and adjusted to the specified pH using 1 M NaOH or 1 M HCl before sterilisation. Unless otherwise stated, media were sterilised by autoclaving at 121°C for 15 min. The compositions of the media (per litre) were as follows:

CAS Detection Medium: Chrome Azurol S (60.5 mg), hexadecyltrimethylammonium bromide (72.9 mg), FeCl_3_·6H_2_O (2.65 mg), NaH_2_PO_4_·2H_2_O (295.25 mg), Na_2_HPO_4_·12H_2_O (1.21 g), NH_4_Cl (125 mg), KH_2_PO_4_ (37.5 mg), NaCl (62.5 mg), and agar (9.0 g), pH 6.8 ± 0.1. Organic Phosphate Solubilising Medium: Glucose (10.0 g), (NH_4_)_2_SO_4_ (0.5 g), yeast extract (0.5 g), NaCl (0.3 g), KCl (0.3 g), MgSO_4_ (0.3 g), FeSO_4_ (0.03 g), MnSO_4_ (0.03 g), lecithin (0.2 g), CaCO_3_ (1.0 g), and agar (15.0 g), pH 7.0–7.5. Inorganic Phosphate Solubilising Medium: Glucose (10.0 g), (NH_4_)_2_SO_4_ (0.5 g), yeast extract (0.5 g), NaCl (0.3 g), KCl (0.3 g), MgSO_4_ (0.3 g), FeSO_4_ (0.03 g), MnSO_4_ (0.03 g), Ca_3_(PO_4_)_2_ (5.0 g), and agar (15.0 g), pH 7.0–7.5. Nitrogen‐Free Medium: Mannitol (10.0 g), KH_2_PO_4_ (0.2 g), MgSO_4_·7H_2_O (0.2 g), NaCl (0.2 g), CaSO_4_·2H_2_O (0.2 g), CaCO_3_ (5.0 g), and agar (20.0 g), pH 7.0–7.2. Silicate Bacterial Medium: Sucrose (5.0 g), MgSO_4_ (0.5 g), CaCO_3_ (0.1 g), Na_2_HPO_4_ (2.0 g), FeCl_3_ (0.005 g), glass powder (1.0 g, < 75 µm), and agar (15.0 g), pH 7.0 ± 0.1.

### 2.7 Indoor Pot Experiment

Surface‐sterilised alfalfa seeds were sown in plastic pots containing 300 g substrate (soil:vermiculite = 3:1, v/v). A completely randomised design was implemented with two sub‐experiments: (i) Bacterial inoculation; and (ii) Root exudate application. The inoculation trial comprised 12 treatment combinations with 12 replicates each, totalling 144 formal experimental units. The root exudate trial included 108 pots with identical replication. To ensure sufficient valid replicates, 126 additional pots were initially planted as reserves; non‐germinated plants and individuals with heights deviating from the population mean were discarded 13 days after sowing. All pots were maintained in a greenhouse under controlled conditions: 16 h photoperiod (8‐h dark) with a photosynthetically active radiation of 300 μmol m^−2^ s^−1^, 23°C–28°C daytime temperature, and 60%–70% relative humidity. Optimal soil moisture was maintained through periodic supplementation with sterile deionised water, avoiding waterlogging.

Treatments commenced 14 days after sowing. For bacterial inoculation: target strains were cultured in LB broth at 28°C and 180 rpm for 12 h, centrifuged (8000 × *g*, 10 min) to collect cells, washed twice with sterile physiological saline (0.85% NaCl), and resuspended to ~1.0 × 10^8^ CFU·mL^−1^. Each plant received 1 mL bacterial suspension applied to the rhizosphere; controls received equivalent sterile saline (Bouarrakia et al. 2025). For root exudate treatment: target metabolites were dissolved at 200 μM in sterile deionised water, filtered through 0.22 μm membranes, and 1 mL solution was applied to the rhizosphere every 7 days starting from day 14 (total 3–4 applications). Controls received equivalent sterile deionised water. Plants were harvested 6 weeks after treatment initiation for measurement of growth parameters including shoot height, root length, shoot fresh weight, and root fresh weight.

### 2.8 Seed Germination Experiment

Seed germination assays were conducted under strictly aseptic conditions. Seeds of alfalfa (*Medicago sativa* L.) of uniform size and weight were selected. Surface sterilisation was performed by immersing seeds in 75% (v/v) ethanol for 1 min, followed by 2% (w/v) sodium hypochlorite for 10 min. The seeds were then rinsed five times with sterile deionised water.

Sterilised seeds were placed on sterile filter paper moistened with sterile deionised water in sterile Petri dishes. For inoculation, the suspension of the screened bacterial strain was adjusted to 1 × 10^8^ CFU mL^−1^. A total of 2 mL of the bacterial suspension was added to each plate. The control group was treated with an equal volume of heat‐killed bacterial suspension (autoclaved at 121°C for 20 min). All manipulations were performed in a laminar flow hood.

The dishes were incubated in a growth chamber at 25°C with a 16 h light/8 h dark photoperiod and 70% relative humidity for 10 days. Germination was recorded daily and defined as radicle emergence exceeding 2 mm. Three independent replicates were performed, with 20 seeds per plate. Contaminated dishes were identified and excluded from data analysis to ensure reliability.

### 2.9 Biofilm Formation Assay

Biofilm formation capacity was quantified using a microtiter 96‐well plate method (Jin et al. 2024). Briefly, overnight‐cultured bacterial suspensions were diluted 1:100 in LB broth, and 100 μL aliquots were transferred to sterile 96‐well flat‐bottom polystyrene microplates. Plates were incubated statically at 28°C in darkness for 48 h to facilitate biofilm development. Following incubation, planktonic cells were gently aspirated, and wells were washed three times with sterile P‐buffered saline (PBS, pH 7.4). Adherent biofilms were fixed with 150 μL methanol per well for 15 min, air‐dried, and stained with 150 μL of 0.1% (w/v) crystal violet solution for 20 min at room temperature. Unbound dye was removed through rinsing with physiological saline, and plates were air‐dried completely. Bound crystal violet was solubilised in 200 μL of 96% ethanol per well, and absorbance was measured at 600 nm using a microplate reader (Thermo Fisher, Dreieich, Germany) to quantify biofilm biomass. Each strain was tested with six technical replicates per strain and three independent experiments.

### 2.10 Statistical Analyses

Statistical analyses were performed using R (v4.1.0; http://www.r-project.org/). Microbial diversity analyses employed the vegan package. Prior to ANOVA, data were evaluated for normality using the Shapiro–Wilk test and homogeneity of variance using Bartlett's test. Differences among treatments were evaluated using *t*‐tests or ANOVA as appropriate. Graphical outputs were generated using a variety of packages: Figures 4A–D and 5A,B,D were generated using the online platform at http://www.cloud.biomicroclass.com/CloudPlatform. Figures 5E and 6A were visualised using Gephi (University of Lyon, Lyon, France). Figure 9 was created using Microsoft PowerPoint (Microsoft, Redmond, WA, USA), and all other figures were generated using OriginPro 2021 (OriginLab, Northampton, MA, USA).

## 3 Results

### 3.1 Screening of High‐ and Low‐Yielding Alfalfa Cultivars and Their Effects on Soil Physicochemical Properties

To characterise yield variation among alfalfa cultivars, per‐plant fresh biomass was quantified for 95 cultivars across two consecutive growing seasons under field conditions. Based on cumulative fresh biomass, eight high‐yielding cultivars (G1–G8: Xinmu No.1, Xinjiang Daye, 55v12, Gongnong No.5, Zhongmu No.1, 54vo9, Kamble, Victoria) and eight low‐yielding cultivars (L1–L8: WL656HQ, WL525HQ, A23, A8, 5262, A20, A28, Ghillie) were selected for further analysis. Over the 2‐year period, cumulative fresh biomass per plant in high‐yielding groups was 90.30% greater than that of the low‐yielding group (Figure 1B).

**Figure 1:**
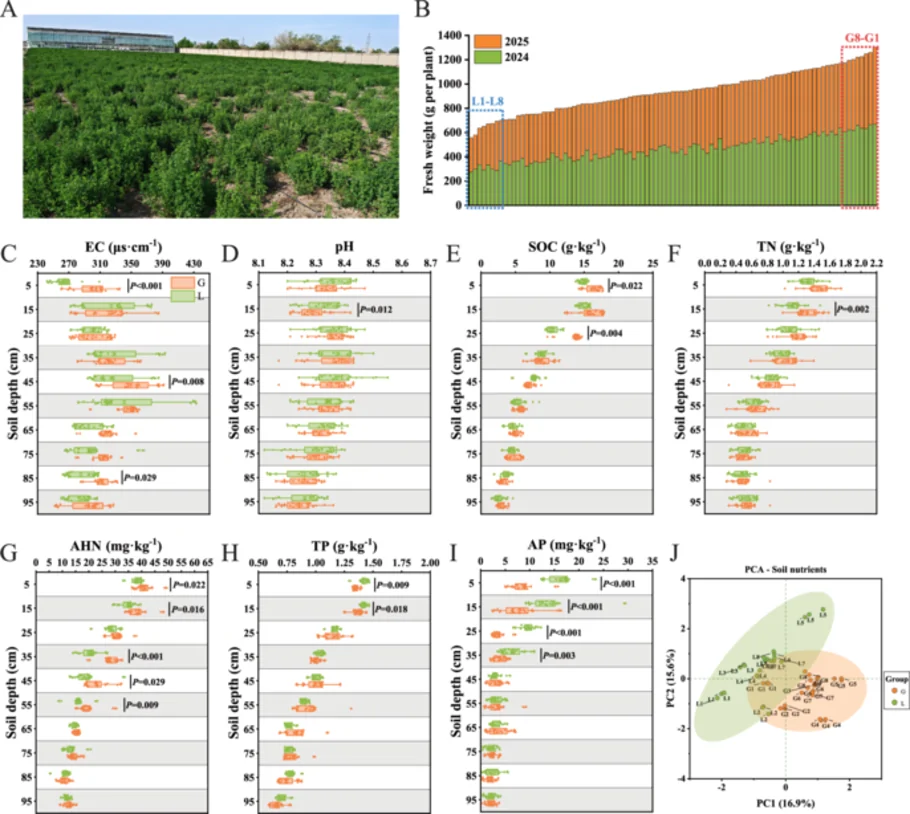
Screening of alfalfa varieties with contrasting yields and comparison of soil physicochemical properties. G, high‐yielding alfalfa groups; L, low‐yielding alfalfa groups. EC, electrical conductivity; SOC, soil organic carbon; TN, total nitrogen; AHN, alkaline hydrolyzable nitrogen; TP, total phosphorus; AP, available phosphorus. (A) Field trial for alfalfa variety screening in the eighth year of continuous cultivation. (B) Selection of high‐ and low‐yielding alfalfa groups. (C–I) Effects of alfalfa yield groups on EC (C), pH (D), SOC (E), TN (F), AHN (G), TP (H), and AP (I) across the 0–100 cm soil profile. (J) Principal component analysis of soil nutrients in fields planted with contrasting‐yield alfalfa groups. *p* < 0.05 indicates significant differences between treatments.

Long‐term cultivation of alfalfa cultivars with contrasting yield performance resulted in distinct soil physicochemical profiles across soil depths (Figure 1J). Soil electrical conductivity was significantly higher under high‐yielding cultivars in the 0–10 cm, 40–50 cm, and 80–90 cm soil layers, by 13.93%, 7.06%, and 9.20%, respectively (*p* < 0.05; Figure 1C). Soil pH in the 10–20 cm layer was slightly but significantly lower (0.47%) under high‐yielding cultivars when compared to the low‐yield cultivars (*p* < 0.05; Figure 1D). Within the 0–40 cm profile, SOC content was generally greater under high‐yielding cultivars, with significant differences observed in the 0–10 cm and 20–30 cm layers (*p* < 0.05; Figure 1E). Total N content in the 10–20 cm layer was 16.01% higher under high‐yielding cultivars (*p* < 0.05; Figure 1F), and AHN content was significantly elevated in both surface (0–20 cm; 8.37%–11.69%) and deeper (30–60 cm; 16.51%–24.42%) soil layers (Figure 1G). In contrast, TP and AP content in the 0–20 cm layers were lower under high‐yielding cultivars than under low‐yielding cultivars (Figure 1H‐I).

### 3.2 High‐Yielding Alfalfa Promotes the Transformation of Soil Stable Phosphorus Into Moderately Labile and Labile Fractions

To further assess the effects of cultivar yield performance on soil P dynamics, soil P fractions were quantified across soil depths. High‐yielding alfalfa significantly reduced the proportion of stable P in the 0–20 cm and 30–40 cm layers (Figure 2G–K). Concurrently, moderately labile P increased by 1.48%–3.34% (Figure 2D–F,J,K). In the 20–30 cm layer, an increase in labile P (0.21%) was observed, coinciding with a decline in moderately labile P (Figure 2G,K).

**Figure 2:**
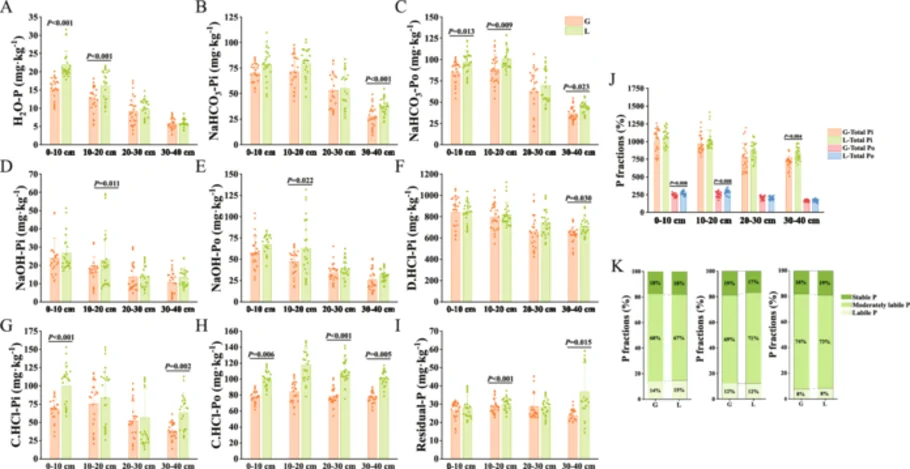
Effects of alfalfa cultivars differing in yield on soil phosphorus fractions. (A–I): Effects on soil H_2_O‐P (A), NaHCO_3_‐Pi (B), NaHCO_3_‐Po (C), NaOH‐Pi (D), NaOH‐Po (E), D. HCl‐Pi (F), C. HCl‐Pi (G), C. HCl‐Po (H), and Residual‐P (I). (J) Effects on soil organic and inorganic phosphorus pools. (K) Effects on soil stable phosphorus, moderately labile phosphorus, and labile phosphorus fractions. *p* < 0.05 indicates significant differences between treatments. [Color figure can be viewed at wileyonlinelibrary.com]

### 3.3 Root Exudate‐Mediated Growth Promotion Depends on the Presence of Rhizosphere Microorganisms

To disentangle the roles of root exudates and rhizosphere microorganisms in plant growth, a compartmented cultivation system was established, with three distinct belowground interaction treatments: Inter (no barrier, allowing root intermingling and exudate exchange), REE (30 μm mesh barrier permitting exudate diffusion while blocking root contact), and Mono (fully isolated compartments preventing all physical and chemical interactions). The experiment was conducted over two consecutive harvests to ensure sufficient root development. Across both cultivars, plant height and fresh mass were significantly greater in non‐sterile soil than in sterilised soil. Under non‐sterile conditions, the high‐yielding cultivar ‘Xinmu No.1’ consistently exhibited significantly higher shoot fresh mass than the low‐yielding cultivar ‘WL656HQ’. For ‘WL656HQ’, plant height under Inter and REE treatments was significantly greater than under Mono in the first harvest (Figure 3D), while both shoot fresh mass and plant height were significantly enhanced under Inter and REE when compared to Mono during the second harvest (Figure 3C,E). In contrast, under sterilised soil conditions, no significant differences in shoot fresh mass or plant height were observed among Inter, REE, and Mono treatments for ‘WL656HQ’ (Figure 3H–K).

**Figure 3:**
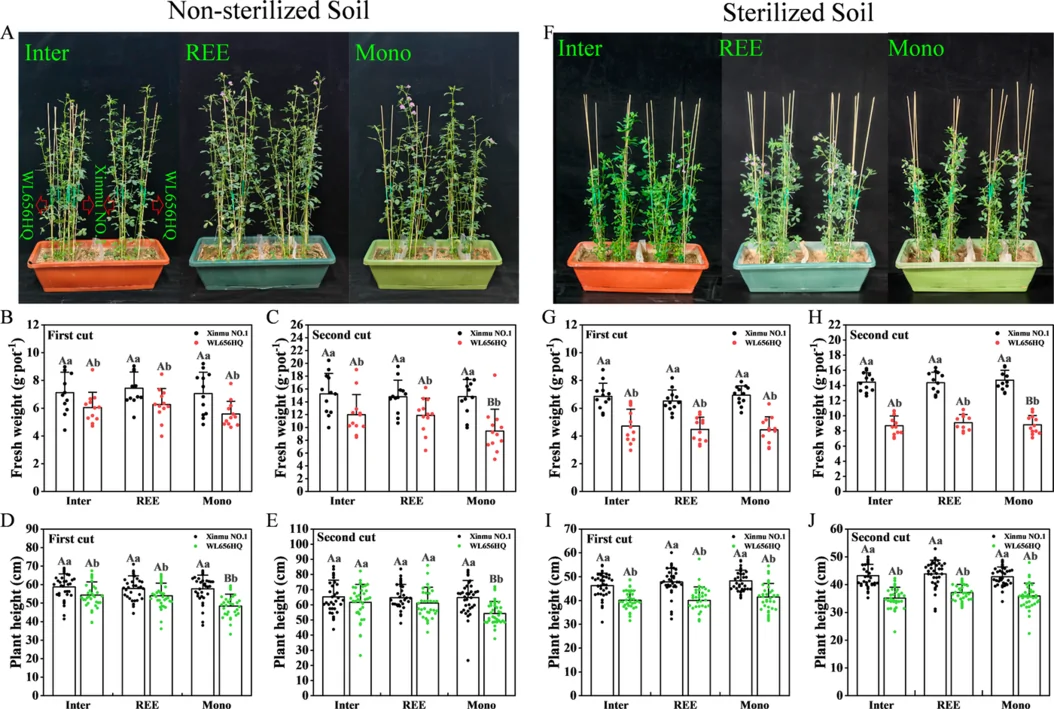
Root exudate‐mediated interactions among alfalfa cultivars with differing yields under sterilised and non‐sterilised conditions. The belowground partitioned cultivation system included three treatments: Inter (no barrier, allowing root intermingling and exudate exchange), REE (30 μm mesh barrier permitting root exudate exchange), and Mono (complete physical isolation preventing all physical and chemical interactions). (A) and (F) show the growth status of alfalfa in the belowground partitioned cultivation experiment under non‐sterilised and sterilised soil conditions, respectively. (B) and (C) represent shoot fresh weights of the first and second harvests under non‐sterilised conditions. (D) and (E) indicate plant heights of alfalfa during the first and second harvests under non‐sterilised conditions. (H) and (I) display shoot fresh weights of the first and second harvests under sterilised conditions. (J) and (K) show plant heights of alfalfa during the first and second harvests under sterilised conditions. Different uppercase letters denote significant differences among partitioning treatments within the same cultivar; different lowercase letters indicate significant differences among cultivars under the same partitioning treatment. [Color figure can be viewed at wileyonlinelibrary.com]

### 3.4 High‐Yielding Alfalfa Alters the Rhizosphere Metabolic Profile by Enriching Key Metabolites

Non‐targeted metabolomic profiling revealed clear differences in root exudate composition between high‐yield and low‐yield treatments. At the metabolite class level, significant differences were observed in lipids and lipid‐like molecules, organic acids and derivatives, benzenoids, and organic N compounds (Figure 4A). Principal component analysis (PCA) showed distinct separation between treatments, with PC1 and PC2 explaining 36.4% and 6.6% of total variance, respectively (Figure 4B). KEGG pathway enrichment analysis indicated that metabolites upregulated in the high‐yield treatment were significantly associated with pathways related to energy metabolism and stress response, including C fixation in photosynthetic organisms, biosynthesis of nucleotide sugars, and sulphur metabolism (Figure 4C). In total, 195 metabolites were significantly upregulated, and 88 were downregulated in the high‐yield treatments (Figure 4D). Among these, maltopentaose, indole‐3‐acetic acid, maltotetraose, acetic acid, taurine, N‐acetyl‐L‐leucine, hydroxybutyric acid, and asparagine were significantly more abundant under high‐yield conditions (*p* < 0.05; Figure 4E).

**Figure 4:**
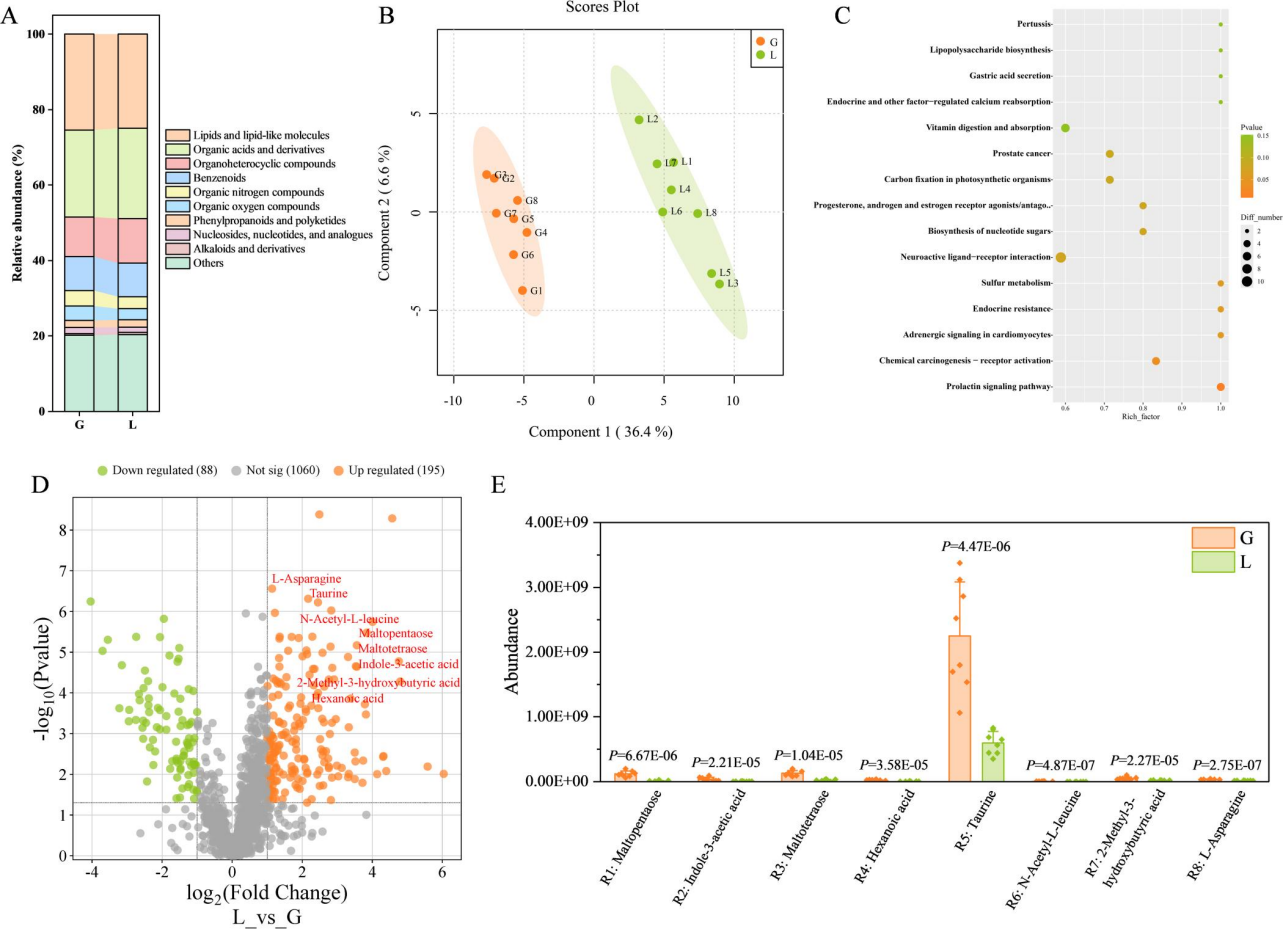
Comparison of root exudate composition between high‐yield and low‐yield alfalfa based on metabolomics. (A) Relative abundance distribution of differential metabolites in high‐yield versus low‐yield treatments. (B) PLS‐DA score plot of root exudates. (C) KEGG metabolic pathways significantly enriched by differential metabolites. (D) Volcano plot of differential metabolites. (E) Comparison of relative abundances of key functionally relevant differential metabolites between groups. [Color figure can be viewed at wileyonlinelibrary.com]

### 3.5 High‐Yielding Alfalfa Enriches *Ensifer*, *Pseudomonas*, and *Bacillus* and Reshapes Rhizosphere Microbial Networks

Analysis of rhizosphere bacterial communities revealed no significant difference in alpha diversity, as indicated by Shannon index or Pielou's evenness indices, between high‐ and low‐yield treatments (*p* > 0.05; Figure 5A). However, PCoA based on Bray‐Curtis dissimilarity showed clear separation between treatments at the ASV level, with PC1 and PC2 explaining 21% and 15% of the total variation, respectively (Figure 5B). Several ASVs were strongly associated with cultivar yield performance, including *Ensifer* ASV77, *Pseudomonas* ASV161, and *Bacillus* ASV21. The relative abundances of these genera (*Ensifer*, *Pseudomonas*, and *Bacillus*) were significantly higher under high‐yield treatment (Figure 5C). Although, at the phylum level, *Acidobacteria*, *Pseudomonadota*, *Actinomycetota*, and *Gemmatimonadota* were dominant in both treatments, their relative abundances differed markedly (Figure 5D). Co‐occurrence network analysis showed that the high‐yield treatment network consisted of 174 nodes and 2207 edges, which exceeded the node and edge counts of the low‐yield treatment. Within the high‐yield network, *Acidobacteria* and *Actinomycetota* exhibited higher connectivity and centrality values compared to the low‐yield network (Figure 5E).

**Figure 5:**
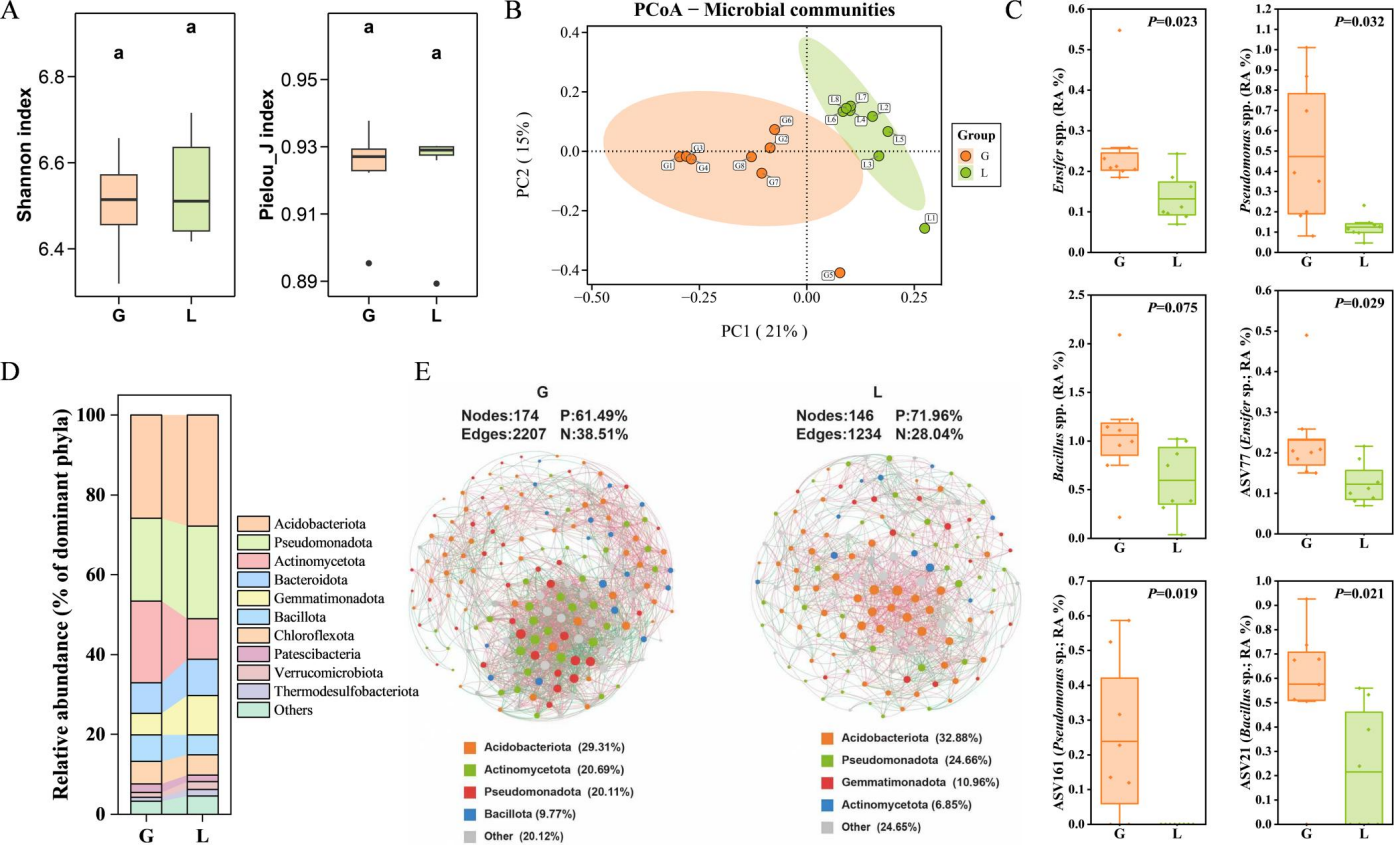
Bacterial community composition in the rhizosphere soil of alfalfa cultivars with differing yields. (A) Comparison of Shannon and Pielou's evenness indices between high‐yield and low‐yield treatments. (B) Principal coordinates analysis (PCoA) based on Bray‐Curtis distance. (C) Relative abundances (RA) of *Ensifer*, *Pseudomonas*, *Bacillus*, ASV77, ASV161, and ASV21 as determined by amplicon sequencing. (D) Differences in bacterial community composition at the phylum level. (E) Comparison of interspecies interaction networks among rhizosphere microbes. [Color figure can be viewed at wileyonlinelibrary.com]

### 3.6 Association Among Root Exudates, Soil Bacteria, and Soil Physicochemical Properties

A multi‐omics co‐occurrence network integrating root metabolites, bacterial taxa, and soil physicochemical properties was constructed to examine rhizosphere interactions under high‐yield conditions (Figure 6A). The network comprised 86 nodes and 702 edges, with positive correlation accounting for 56.7% of total interaction. Modular analysis identified three distinct modules, with Module 2 emerging as the central hub. This module was enriched in key PGPR genera (*Ensifer*, *Pseudomonas*, and Bacillus), differential metabolites (e.g., maltopentaose, indole‐3‐acetic acid, maltotetraose), and soil fertility indicators (SOC, TN, AHN, and moderately labile P). Linear correlation analysis showed that TN and AHN were positively correlated with *Pseudomonas*, whereas TP and AP were negatively correlated with *Bacillus* (Figure 6B). Differential root exudates were positively correlated with TN, AHN, and moderately labile P, but negatively correlated with readily available AP and labile P (Figure 6C). All three key genera (*Ensifer*, *Pseudomonas*, and *Bacillus*) displayed significant positive correlations with multiple differential root exudates (Figure 6D).

**Figure 6:**
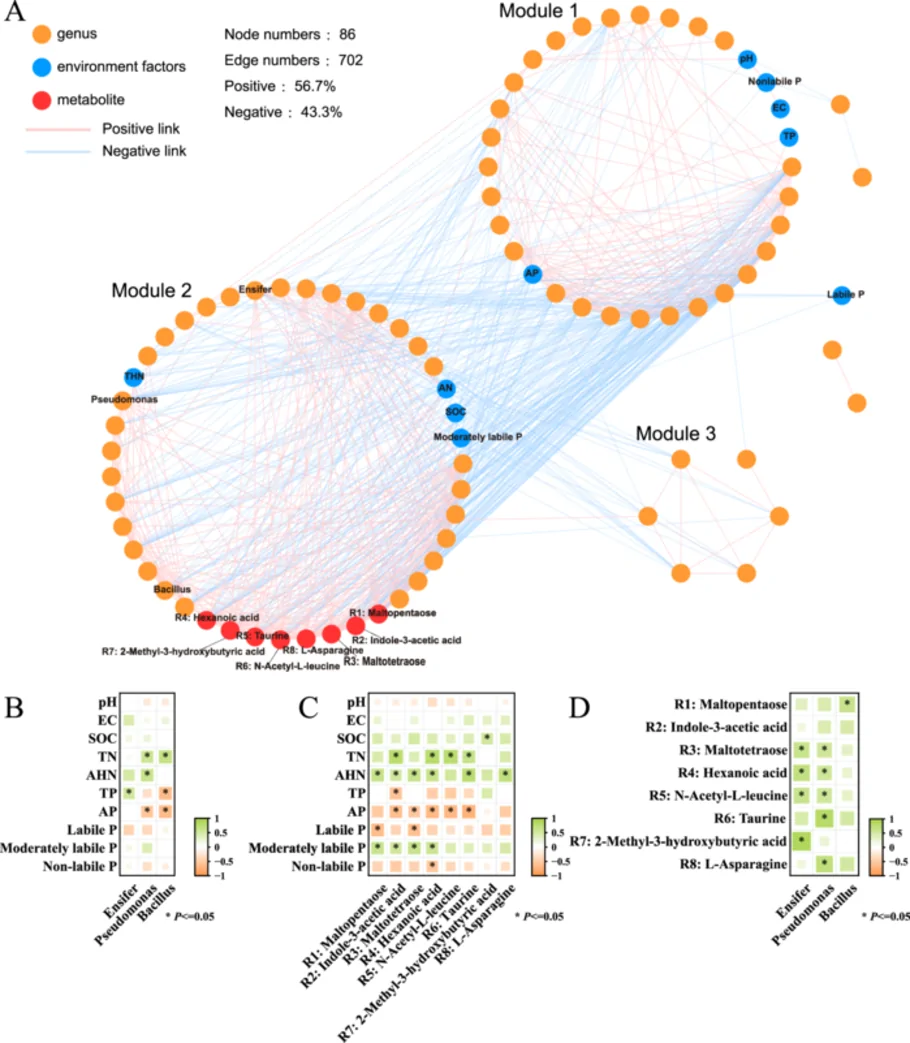
Relationships among bacteria, root exudates, and soil physicochemical properties. EC, electrical conductivity; SOC, soil organic carbon; TN, total nitrogen; AHN, alkaline hydrolyzable nitrogen; TP, total phosphorus; AP, available phosphorus. (A) Multi‐omics co‐occurrence network of root metabolites–soil bacteria–soil physicochemical properties; (B) Pearson correlation analysis between soil physicochemical properties and soil bacteria; (C) Pearson correlation analysis between soil physicochemical properties and root exudates; (D) Pearson correlation analysis between soil bacteria and root exudates. [Color figure can be viewed at wileyonlinelibrary.com]

### 3.7 Screening and Functional Evaluation of Efficient Plant Growth‐Promoting Rhizobacteria Strains

From rhizosphere soil samples associated with 16 alfalfa cultivars, 605 culturable bacterial strains were isolated. Following functional screening, 83 strains exhibiting plant growth‐promoting potential were obtained. Phylogenetic analysis based on 16S rRNA gene sequencing identified multiple strains belonging to key PGPR genera, including 8 *Ensifer*, 6 *Pseudomonas*, and 2 *Bacillus* strains (Figure 7A,B).

**Figure 7:**
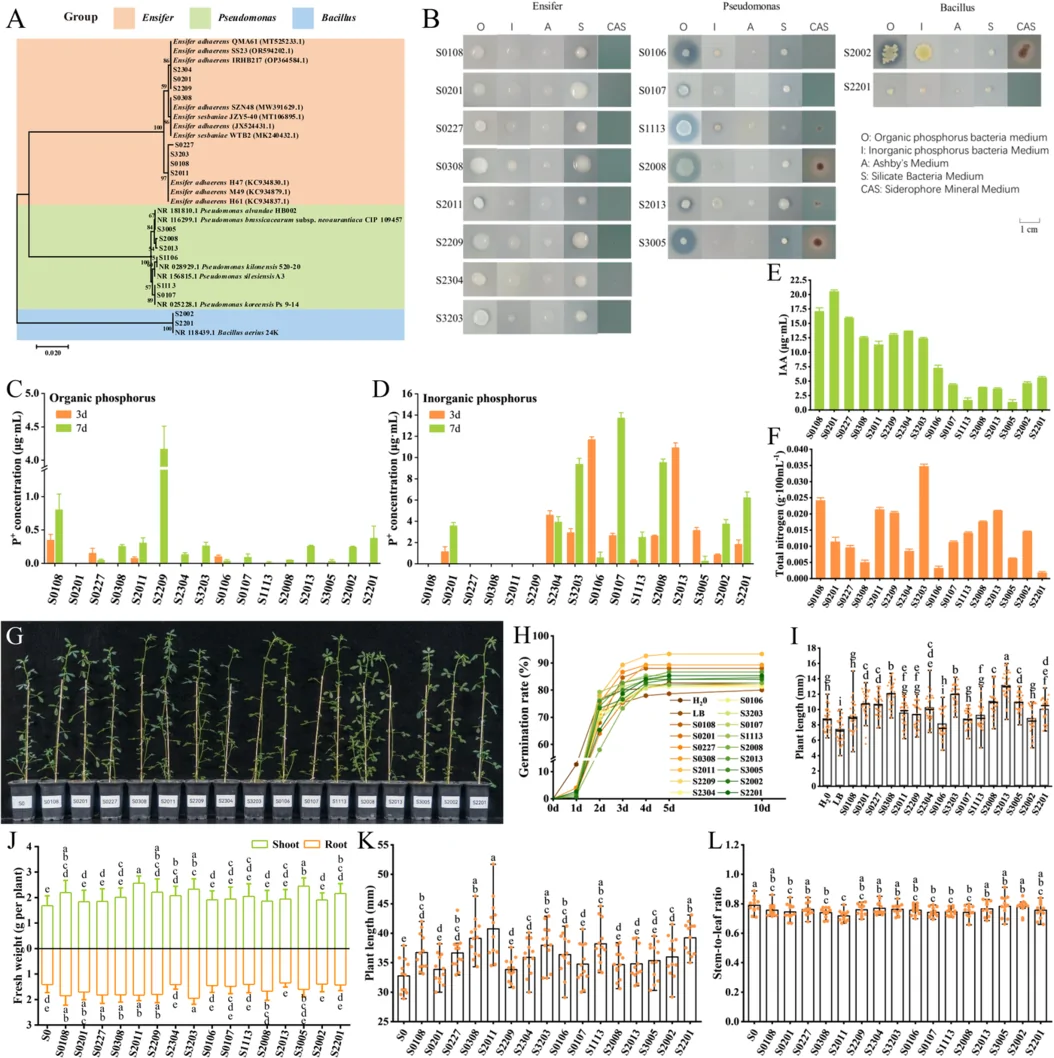
Screening of *Ensifer*, *Pseudomonas*, and *Bacillus* and phenotypic evaluation in greenhouse pot experiments. (A) Phylogenetic tree. (B) Growth performance of rhizosphere plant growth‐promoting bacteria on different selective media. (C, D) Phosphorus‐solubilising capacity of rhizosphere plant growth‐promoting bacteria on organic and inorganic phosphorus‐solubilising media, respectively. (E) IAA production by rhizosphere plant growth‐promoting bacteria. (F) Nitrogen‐fixing capacity of rhizosphere plant growth‐promoting bacteria on nitrogen‐free medium. (G) Growth status of alfalfa inoculated with rhizosphere plant growth‐promoting bacteria. (H, I) Effects of rhizosphere plant growth‐promoting bacteria on alfalfa seed germination rate and seedling height, respectively. (J–L) Effects of rhizosphere plant growth‐promoting bacteria on aboveground and belowground biomass, plant height, and stem‐to‐leaf ratio of alfalfa, respectively. Different lowercase letters indicate significant differences among treatments. [Color figure can be viewed at wileyonlinelibrary.com]

Further assays revealed that all tested strains exhibited N fixation potential and IAA production. By day 3, 4 strains showed organic P‐solubilising activity while 11 strains exhibited inorganic P‐solubilising activity, and by day 7, 14 strains showed organic P‐solubilising ability and 10 strains exhibited inorganic P‐solubilising ability (Figure 7C–F). Seed germination assays showed that 9 strains significantly promoted alfalfa seed germination rate (Figure 7H) and increased seedling height (*p* < 0.05; Figure 7I). In a 45‐day pot experiment (Figure 7G), seven strains (S0108, S2011, S2209, S2304, S3203, S3005, and S2201) significantly enhanced aboveground biomass (Figure 7J), while ten strains (S0108, S0227, S0308, S2011, S2304, S3203, S0106, S1113, S2002, and S2201) significantly increased plant height when compared with the control (Figure 7K). Six strains (S0201, S0308, S2011, S0107, S1113, and S2008) significantly reduced the stem‐to‐leaf ratio when compared to controls (Figure 7L).

### 3.8 Differential Root Exudates Enhance Plant Growth‐Promoting Rhizobacteria Growth, Biofilm Formation and Plant Performance

Based on pot inoculation results, *Ensifer* strain S2011, *Pseudomonas* strain S3005, and *Bacillus* strain S2201 were selected to evaluate responses to root exudates from different sources (R1–R8). Several exudate treatments significantly promoted bacterial growth, with R1, R3, R4, R5, R6, and R8 enhancing growth of S2011; R1, R4, R5, and R6 enhanced the growth of S3005; and R1, R3, R5, R6, and R8 enhanced growth of S2201 (Figure 8F). Plate‐counting assays confirmed enhanced bacterial colonisation under multiple exudate treatments (R1, R3, R5, R6, and R8) (Figure 8E). Biofilm assays showed that R1, R5, and R8 significantly enhanced biofilm formation by S2011; R1, R5, R6, and R8 significantly enhanced biofilm formation by S3005; and R1, R3, R5, R6, and R8 significantly enhanced biofilm formation by S2201 (Figure 8G).

**Figure 8:**
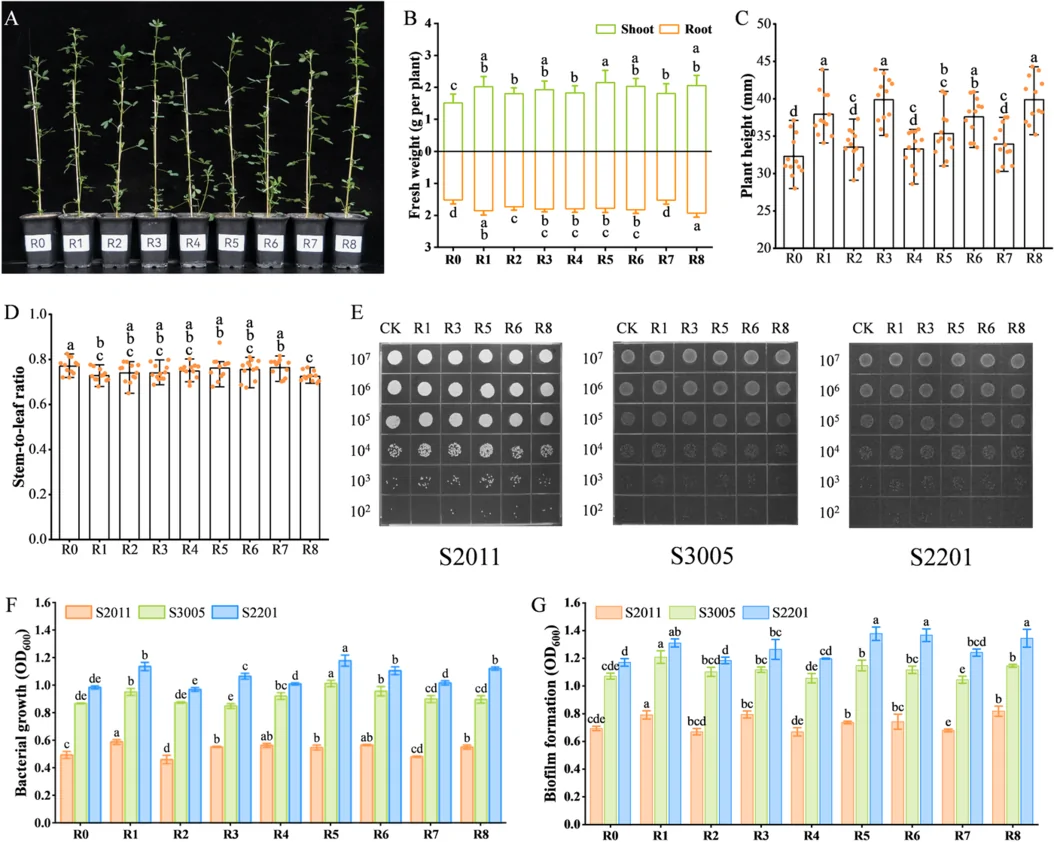
Effects of key differential root exudates on bacterial growth and biofilm formation. R1, R2, R3, R4, R5, R6, R7, and R8 represent maltopentaose, indole‐3‐acetic acid, maltotetraose, hexanoic acid, taurine, N‐acetyl‐L‐leucine, 2‐Methyl‐3‐hydroxybutyric acid, and L‐Asparagine, respectively. (A) Growth status of alfalfa under supplementation with root exudates. (B) Aboveground and belowground biomass of alfalfa under root exudate treatments. (C, D) Seedling height and stem‐to‐leaf ratio of alfalfa under root exudate treatments, respectively. (E) Effects of root exudates on the growth of rhizosphere plant growth‐promoting bacteria at different concentrations. (F, G) Optical density (OD) values reflecting the growth and biofilm formation of rhizosphere plant growth‐promoting bacteria following treatment with different root exudates. Different lowercase letters indicate significant differences among treatments. [Color figure can be viewed at wileyonlinelibrary.com]

**Figure 9:**
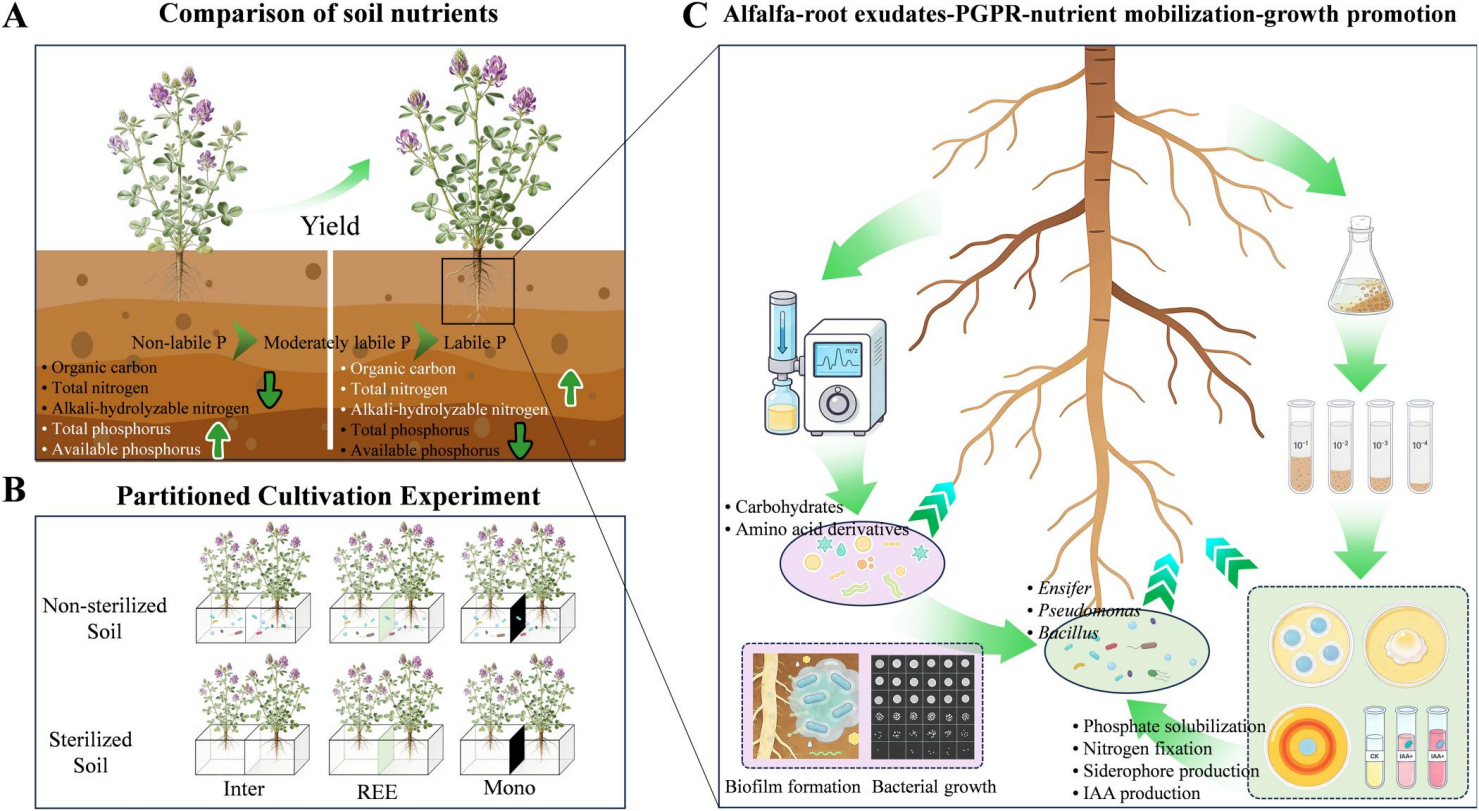
Schematic diagram illustrating the mechanism by which root exudates mediate plant growth‐promoting rhizobacteria (PGPR) recruitment and nutrient transformation. (A) Comparison of soil nutrients between high‐yield and low‐yield alfalfa fields. (B) Partitioned cultivation experiment. (C) Model of alfalfa root exudates ‐ plant growth‐promoting rhizobacteria ‐ nutrient mobilisation ‐ growth promotion. [Color figure can be viewed at wileyonlinelibrary.com]

In the pot experiment, all root exudate treatments except for R7 significantly increased aboveground biomass, belowground biomass, and plant height of alfalfa (*p* < 0.05; Figure 8A–C). Notably, R1 and R8 significantly reduced the stem‐to‐leaf ratio compared with the control (*p* < 0.05; Figure 8D).

## 4 Discussion

Global agricultural intensification has relied heavily on widespread adoption of high‐yielding crop varieties to increase food, fibre, and fuel production, yet these systems are often assumed to accelerate soil nutrient depletion and ecosystem degradation (Matson et al. 1997). This assumption largely reflects a linear agronomic paradigm in which biomass production is equated with proportionally greater nutrient extraction, with limited consideration of biologically mediated feedbacks operating within plant–soil‐microbe systems (Abanda et al. 2011). While it is well established that high‐yielding crops remove more nutrients from the soil due to their greater biomass (Li et al. 2020), our finding challenges the expectation that this inevitably leads to a long‐term decline in soil fertility. In that, when compared with low‐yielding alfalfa varieties, continuous cultivation of high‐yielding alfalfa over multiple years did not lead to soil nutrient deficit, but instead, we observed increased SOC and AHN. Although TP content did not increase, the system facilitated as shift from stable P pools towards moderately labile and labile fractions, indicating enhanced biological P activation rather than a net depletion. Collectively, these results demonstrate that a high‐yielding alfalfa system can ‘self‐reinforce’ nutrient dynamics, whereby productivity is maintained not through mining of soil reserves, but through stimulation of ‘internal’ and/or proximal nutrient cycles. We argue that our finding reframes high‐yielding crops, not as an ecological liability, but as a potential driver of beneficial plant‐soil feedbacks mediated through the rhizosphere (Liang et al. 2026; Raza et al. 2024).

An increasing body of evidence supports the view that plants are not passive nutrient consumers (Ling et al. 2022; Zhou et al. 2023) but are active engineers of their rhizosphere environments. Root exudates represent a central mechanism in this process, linking plant C metabolism with microbial community assembly and function. Plant species have been estimated to release between 5% and 21% of photosynthetically fixed C into the rhizosphere as root exudates (Sasse et al. 2018; Massalha et al. 2017), comprised of sugars, amino acids, organic acids, phytohormones, and secondary metabolites. These compounds not only supply C and energy sources for microbes but also function as signalling molecules that selectively modulate microbial activity and community structure (Xu et al. 2025).

Our partitioned cultivation experiment provides direct evidence that root exudates exert their growth‐promoting effects primarily through microbial mediation. In that, root exudates enhanced alfalfa growth only in non‐sterilised soils, while no effect was observed under sterile conditions. Suggesting that root exudates do not function simply as supplementary nutrients, but instead regulate rhizosphere processes by shaping microbial composition and activity. We argue that the benefits of root exudation are in reality ‘emergent’ (and undervalued) properties of plant‐microbial composition and activity, rather than intrinsic chemical effects, and warrant further investigation.

Metabolomic analysis revealed that high‐yielding alfalfa releases a distinct suite of compounds, including oligosaccharides (e.g., maltopentaose and maltotetraose) and amino acid derivatives (e.g., taurine, N‐acetyl‐L‐leucine, and asparagine), which collectively create ‘chemical niches’ favourable for recognition, colonisation, and persistence of PGPR. These metabolites selectively attract diverse PGPR taxa and promote their proliferation and biofilm formation, indicating a targeted recruitment strategy, rather than a diffuse stimulation of the soil microbiome. This selective enrichment suggests an adaptive mechanism by which high‐yielding plants actively assemble a rhizosphere community aligned and tailored to their physiological demands under sustained production pressure (Zhang et al. 2023).

Consistent with this interpretation, in our study, 16S rRNA gene sequencing revealed that long‐term cultivation of high‐yielding alfalfa resulted in a stable and highly specific rhizosphere microbiome, characterised by significant enrichment of bacterial genera, including *Ensifer*, *Pseudomonas*, and *Bacillus*. These taxa are well documented for their role in N fixation, P solubilisation, phytohormone production, and pathogen suppression (Tournay et al. 2023; Wang et al. 2024b; Yang et al. 2024). In our study, we isolated 83 PGPR strains from the alfalfa rhizosphere, including 16 strains belonging to these dominant genera (*Ensifer*, *Pseudomonas*, and *Bacillus*). Functional assays confirmed that most isolates possessed multiple plant growth‐promoting traits, and pot inoculation experiments demonstrated that individual strains enhanced alfalfa biomass accumulation. However, this enhanced biomass may also indirectly contribute to Ensifer enrichment, as it has been suggested that Ensifer, as a symbiotic rhizobial genus, exists within nodules (Kearsley et al. 2024). Specifically, the high‐biomass root system of high‐yielding alfalfa may provide more infection sites for rhizobia, thereby increasing *Ensifer* abundance within nodules. However, whether this increase in nodule *Ensifer* directly correlates with the observed enrichment of *Ensifer* in the rhizosphere soil remains to be further studied.

Notably, despite their phylogenetic diversity, these enriched taxa displayed a high degree of functional redundancy and complementarity. For example, traits for N fixation, P solubilisation, siderophore production, and IAA synthesis were widely distributed across strains. This functional overlap likely confers resilience to the rhizosphere microbial ‘network’, ensuring stability of nutrient mobilisation processes even under environmental perturbation (Ling et al. 2022). We suggest that this ‘redundancy’ provides a plausible ecological explanation for the sustained productivity observed under long‐term monoculture of high‐yielding alfalfa (Wang et al. 2025c), which reinforces the concept that plants selectively recruit microbial consortia that are optimised for both efficiency and robustness through the secretion of specific signalling molecules.

Together, our results support the existence of a positive feedback loop, where “alfalfa ‐ root exudates ‐ PGPR ‐ nutrient activation ‐ growth promotion”, which underpins sustained high yields. High‐yielding plants allocate photosynthetically fixed C (e.g., organic acids, amino acids, and sugars) to the rhizosphere, stimulating microbial‐mediated processes that enhance N and P availability, thereby reinforcing plant growth and further C inputs belowground. This reciprocal exchange exemplifies cross‐kingdom resource coupling and aligns with emerging evidence from other cropping systems (Sun et al. 2024). Similar feedbacks have been documented in legumes via nodulation‐mediated shifts in rhizosphere pH and redox status (Jaiswal et al. 2018), and in gramineous crops through optimised exudation profiles (Xu et al. 2025), and in systems involving P‐solubilising bacteria and/or arbuscular mycorrhizal fungi, which contribute to both nutrient acquisition and soil structure stability (Kohler et al. 2010; Li et al. 2025).

Furthermore, we suggest that root exudation is not static but highly plastic; it responds dynamically to environmental constraints. In that, plants can alter exudate composition under nutrient limitation or abiotic stress to recruit ‘functionally matched’ microbial partners, for example, increased secretion of coumarins under P deficiency in Arabidopsis [*Arabidopsis thaliana* (L.) Heynh.] (Afridi et al. 2024; Ziegler et al. 2015), or organic acids under heavy metal stress in poplar (*Populus tremula* L.) (Qin et al. 2007). These findings underscore root exudation as a rapid and adaptive ecological strategy through which plants regulate microbial function to meet changing environmental demands/conditions (Sasse et al. 2018).

Despite the clear role of ‘environmental filtering’, host genetic background also exerts strong control over rhizosphere assembly. Whether the key root exudate components identified here are regulated by specific genetic loci, and whether similar metabolic strategies are conserved across other high‐yielding legumes or perennial crops, remains unresolved. Comparative analyses across genotypes differing in yield potential, combined with functional microbiome profiling, will be essential for disentangling the genetic, metabolic, and microbial linkages underlying the “high yield ‐ root exudation behaviour ‐ microbial recruitment” continuum. We argue that these insights could ultimately inform the incorporation of rhizosphere traits into breeding targets/programs.

We suggest that, from an applied perspective, our findings also highlight limitations of current microbial inoculation strategies, which often fail due to poor establishment and context‐dependent performance. In contrast, leveraging endogenous plant exudation pathways to recruit beneficial microbes from ‘native’ soil communities may offer a more durable and ecologically aligned approach. Although still emerging, studies demonstrating target manipulation of root exudation in crops such as rice (*Oryza sativa* L.) and wheat (*Triticum aestivum* L.) provide ‘proof of concept’ mediated for exudate‐mediated microbiome engineering (Kawasaki et al. 2021).

In summary, this study demonstrates that high‐yielding alfalfa sustains long‐term productivity by actively reshaping its rhizosphere through targeted root exudation, selectively enriching multifunctional PGPR that drive N and P transformation. In that, our findings provide mechanistic support for extending plant‐microbe coevolution theory into the design of sustainable, high yield, and resilient agroecosystems. Integrating root exudation traits into breeding and management frameworks may enable the development of a ‘new’ generation of yield‐enhancing strategies, which are grounded in microbiome regulation, and support the transition towards low‐C, resource‐efficient, and resilient agricultural systems.

## 5 Conclusion

This study elucidates a mechanistic framework linking root exudation, selective recruitment of PGPR, and enhanced soil nutrient transformation in the rhizosphere of high‐yielding alfalfa. By integrating long‐term field observations with controlled partitioned cultivation, microbial, and metabolomic analyses, we demonstrate that sustained alfalfa productivity is underpinned by an active, biologically mediated rhizosphere regulatory process rather than by passive nutrient depletion or short‐term input effects. After eight consecutive years of cultivation, high‐yielding alfalfa significantly increased SOC and TN, and promoted the conversion of stable P into moderately labile and labile forms, thereby enhancing soil nutrient supply capacity. Partitioned cultivation experiments confirmed that the growth‐promoting effects of root exudates were contingent on the presence of an active rhizosphere microbiome, indicating that exudate function is primarily driven through microbially mediated processes rather than direct plant uptake. High‐yielding alfalfa continently assembled a PGPR‐enriched microbiome dominated by *Ensifer*, *Pseudomonas*, and *Bacillus*. These taxa exhibited strong functional capacities for N fixation, P solubilisation, and IAA synthesis, which collectively improve nutrient bioavailability and plant growth. Metabolomic profiling further identified specific low‐molecular‐weight compounds, including maltopentaose, maltotetraose, taurine, N‐acetyl‐L‐leucine, and asparagine, which act as chemical cues to attract and stimulate PGPR. These metabolites enhanced bacterial chemotaxis, proliferation, and biofilm formation, thereby facilitating the targeted establishment of functionally beneficial microbial consortia. Collectively, our findings indicate that high‐yielding alfalfa cultivars actively engineer their rhizosphere microbiome through selective exudation, creating a positive feedback loop between microbial nutrient transformation and sustained plant productivity. Our study advances understanding of the rhizosphere mechanisms that support long‐term performance in perennial legume systems and provides a conceptual and mechanistic basis for microbiome‐informed breeding rhizosphere engineering strategies aimed at improving the sustainability of forage production systems under resource‐limited and changing environmental conditions.

## Conflicts of Interest

The authors declare no conflicts of interest.

## Data Availability

The data that support the findings of this study are available from the corresponding author upon reasonable request.

## References

[pce70631-bib-0001] Abanda, P. A. , J. S. Compton , and R. E. Hannigan . 2011. “Soil Nutrient Content, Above‐Ground Biomass and Litter in a Semi‐Arid Shrubland, South Africa.” Geoderma 164: 128–137. 10.1016/j.geoderma.2011.05.015.

[pce70631-bib-0002] Afridi, M. S. , A. Kumar , M. A. Javed , A. Dubey , F. H. V. de Medeiros , and G. Santoyo . 2024. “Harnessing Root Exudates for Plant Microbiome Engineering and Stress Resistance in Plants.” Microbiological Research 279: 127564. 10.1016/j.micres.2023.127564. 38071833

[pce70631-bib-0003] Annicchiarico, P. , B. Barrett , E. C. Brummer , B. Julier , and A. H. Marshall . 2014. “Achievements and Challenges in Improving Temperate Perennial Forage Legumes.” Critical Reviews in Plant Sciences 34: 327–380. 10.1080/07352689.2014.898462.

[pce70631-bib-0004] Bao, S. D. 2000. Soil and Agricultural Chemistry Analysis, 30–82. China Agriculture Press.

[pce70631-bib-0005] Bouarrakia, M. B. , A. Elyemlahi , O. Elgaliou , et al. 2025. “Impact of Bacillus Subtilis and Neobacillus Niacini on Growth and Zinc Uptake in Alfalfa Under Stress.” International Journal of Environmental Science and Technology 22: 14429–14438. 10.1007/s13762-025-06593-0.

[pce70631-bib-0006] Cesari, A. , N. Paulucci , M. López‐Gómez , J. Hidalgo‐Castellanos , C. L. Plá , and M. S. Dardanelli . 2019. “Restrictive Water Condition Modifies the Root Exudates Composition During Peanut‐PGPR Interaction and Conditions Early Events, Reversing the Negative Effects on Plant Growth.” Plant Physiology and Biochemistry 142: 519–527. 10.1016/j.plaphy.2019.08.015. 31450055

[pce70631-bib-0007] Chowdhuri, I. , and S. C. Pal . 2025. “Challenges and Potential Pathways Towards Sustainable Agriculture Crop Production: A Systematic Review to Achieve Sustainable Development Goals (SDGs).” Soil and Tillage Research 248: 106442. 10.1016/j.still.2024.106442.

[pce70631-bib-0008] Gowtham, H. G. , S. B. Singh , N. Shilpa , et al. 2022. “Insight into Recent Progress and Perspectives in Improvement of Antioxidant Machinery Upon PGPR Augmentation in Plants Under Drought Stress: A Review.” Antioxidants 11: 1763. 10.3390/antiox11091763. PMC9495777 36139837

[pce70631-bib-0009] Guppy, C. N. , N. W. Menzies , P. W. Moody , B. L. Compton , and F. P. C. Blamey . 2000. “Analytical Methods and Quality Assurance.” Communications in Soil Science and Plant Analysis 31: 1981–1991. 10.1080/00103620009370556.

[pce70631-bib-0010] Hadidi, M. , J. C. Orellana Palacios , D. J. McClements , M. Mahfouzi , and A. Moreno . 2023. “Alfalfa as a Sustainable Source of Plant‐Based Food Proteins.” Trends in Food Science & Technology 135: 202–214. 10.1016/j.tifs.2023.03.023.

[pce70631-bib-0011] He, F. , S. Chen , Y. Zhang , et al. 2025. “Pan‐Genomic Analysis Highlights Genes Associated With Agronomic Traits and Enhances Genomics‐Assisted Breeding in Alfalfa.” Nature Genetics 57: 1262–1273. 10.1038/s41588-025-02164-8. 40269327

[pce70631-bib-0012] Hedley, M. J. , J. W. B. Stewart , and B. S. Chauhan . 1982. “Changes in Inorganic and Organic Soil Phosphorus Fractions Induced by Cultivation Practices and by Laboratory Incubations.” Soil Science Society of America Journal 46: 970–976. 10.2136/sssaj1982.03615995004600050017x.

[pce70631-bib-0013] Jaiswal, S. K. , J. Naamala , and F. D. Dakora . 2018. “Nature and Mechanisms of Aluminium Toxicity, Tolerance and Amelioration in Symbiotic Legumes and Rhizobia.” Biology and Fertility of Soils 54: 309–318. 10.1007/s00374-018-1262-0. PMC6560468 31258230

[pce70631-bib-0014] Jin, X. , H. Jia , L. Ran , et al. 2024. “Fusaric Acid Mediates the Assembly of Disease‐Suppressive Rhizosphere Microbiota via Induced Shifts in Plant Root Exudates.” Nature Communications 15: 5125. 10.1038/s41467-024-49218-9. PMC11180119 38879580

[pce70631-bib-0015] Kawasaki, A. , P. G. Dennis , C. Forstner , et al. 2021. “Manipulating Exudate Composition From Root Apices Shapes the Microbiome Throughout the Root System.” Plant Physiology 187: 2279–2295. 10.1093/plphys/kiab337. PMC8644255 34618027

[pce70631-bib-0016] Kearsley, J. V. S. , L. M. Sather , and T. M. Finan . 2024. “ *Sinorhizobium* (*Ensifer*) *Meliloti* .” Trends in Microbiology 32: 516–518. 10.1016/j.tim.2024.03.004. 38575429

[pce70631-bib-0017] Kohler, J. , F. Caravaca , and A. Roldán . 2010. “An AM Fungus and a PGPR Intensify the Adverse Effects of Salinity on the Stability of Rhizosphere Soil Aggregates of *Lactuca sativa* .” Soil Biology and Biochemistry 42: 429–434. 10.1016/j.soilbio.2009.11.021.

[pce70631-bib-0018] Li, Y. , L. Lu , Q. Wang , et al. 2025. “Arbuscular Mycorrhizal Fungi Promote Nodulation and N(2) Fixation in Soybean by Specific Root Exudates.” Plant, Cell & Environment 48: 5514–5528. 10.1111/pce.15529. 40195807

[pce70631-bib-0019] Li, Z. , Z. Liu , M. Zhang , et al. 2020. “Long‐Term Effects of Controlled‐Release Potassium Chloride on Soil Available Potassium, Nutrient Absorption and Yield of Maize Plants.” Soil and Tillage Research 196: 104438. 10.1016/j.still.2019.104438.

[pce70631-bib-0020] Liang, W. , X. Huang , Y. Wang , et al. 2026. “Fertilization Improves Tobacco Yield and Quality by Regulating Soil Microorganisms.” Industrial Crops and Products 239: 122453. 10.1016/j.indcrop.2025.122453.

[pce70631-bib-0021] Liao, J. , P. Xia , and Y. Zhang . 2025. “Factors Affecting and Ameliorating Continuous Cropping Obstacles in *P. Notoginseng* .” Applied Soil Ecology 214: 106351. 10.1016/j.apsoil.2025.106351.

[pce70631-bib-0022] Ling, N. , T. Wang , and Y. Kuzyakov . 2022. “Rhizosphere Bacteriome Structure and Functions.” Nature Communications 13: 836. 10.1038/s41467-022-28448-9. PMC8837802 35149704

[pce70631-bib-0024] Liu, Z. , Z. Wu , F. Tian , et al. 2023. “Phosphate‐Solubilizing Microorganisms Regulate the Release and Transformation of Phosphorus in Biochar‐Based Slow‐Release Fertilizer.” Science of the Total Environment 869: 161622. 10.1016/j.scitotenv.2023.161622. 36649772

[pce70631-bib-0025] Massalha, H. , E. Korenblum , D. Tholl , and A. Aharoni . 2017. “Small Molecules Below‐Ground: The Role of Specialized Metabolites in the Rhizosphere.” Plant Journal 90: 788–807. 10.1111/tpj.13543. 28333395

[pce70631-bib-0026] Matson, P. A. , W. J. Parton , A. G. Power , and M. J. Swift . 1997. “Agricultural Intensification and Ecosystem Properties.” Science 277: 504–509. 10.1126/science.277.5325.504. 20662149

[pce70631-bib-0027] Oulad Ziane, S. , Z. Imehli , Z. El Alaoui Talibi , S. Ibnsouda Koraichi , A. Meddich , and C. El Modafar . 2024. “Biocontrol of Tomato *Verticillium* Wilt Disease by Plant Growth‐Promoting Bacteria Encapsulated in Alginate Extracted From Brown Seaweed.” International Journal of Biological Macromolecules 276: 133800. 10.1016/j.ijbiomac.2024.133800. 38996895

[pce70631-bib-0028] Pantigoso, H. A. , Y. He , M. J. DiLegge , and J. M. Vivanco . 2020. Methods for Root Exudate Collection and Analysis. Springer US. 10.1007/978-1-0716-1040-4_22 33161555

[pce70631-bib-0030] Qin, R. , Y. Hirano , and I. Brunner . 2007. “Exudation of Organic Acid Anions From Poplar Roots After Exposure to Al, Cu and Zn.” Tree Physiology 27: 313–320. 10.1093/treephys/27.2.313. 17241973

[pce70631-bib-0031] Raza, W. , G. Jiang , N. Eisenhauer , et al. 2024. “Microbe‐Induced Phenotypic Variation Leads to Overyielding in Clonal Plant Populations.” Nature Ecology & Evolution 8: 392–399. 10.1038/s41559-023-02297-1. 38195997

[pce70631-bib-0032] Saraf, M. , U. Pandya , and A. Thakkar . 2014. “Role of Allelochemicals in Plant Growth Promoting Rhizobacteria for Biocontrol of Phytopathogens.” Microbiological Research 169: 18–29. 10.1016/j.micres.2013.08.009. 24176815

[pce70631-bib-0033] Sasse, J. , E. Martinoia , and T. Northen . 2018. “Feed Your Friends: Do Plant Exudates Shape the Root Microbiome?” Trends in Plant Science 23: 25–41. 10.1016/j.tplants.2017.09.003. 29050989

[pce70631-bib-0034] Sellick, C. A. , R. Hansen , G. M. Stephens , R. Goodacre , and A. J. Dickson . 2011. “Metabolite Extraction From Suspension‐Cultured Mammalian Cells for Global Metabolite Profiling.” Nature Protocols 6: 1241–1249. 10.1038/nprot.2011.366. 21799492

[pce70631-bib-0035] Sharma, I. , S. Sharma , V. Sharma , et al. 2024. “PGPR‐Enabled Bioremediation of Pesticide and Heavy Metal‐Contaminated Soil: A Review of Recent Advances and Emerging Challenges.” Chemosphere 362: 142678. 10.1016/j.chemosphere.2024.142678. 38908452

[pce70631-bib-0036] Sun, X. , C. Jiang , Y. Guo , et al. 2024. “Suppression of *OsSAUR2* Gene Expression Immobilizes Soil Arsenic Bioavailability by Modulating Root Exudation and Rhizosphere Microbial Assembly in Rice.” Journal of Hazardous Materials 473: 134587. 10.1016/j.jhazmat.2024.134587. 38772107

[pce70631-bib-0037] Tian, D. , Y. Xiang , B. Wang , et al. 2018. “Cropland Abandonment Enhances Soil Inorganic Nitrogen Retention and Carbon Stock in China: A Meta‐Analysis.” Land Degradation & Development 29: 3898–3906. 10.1002/ldr.3137.

[pce70631-bib-0038] Tournay, R. J. , A. Firrincieli , S. S. Parikh , D. M. Sivitilli , and S. L. Doty . 2023. “Effect of Arsenic on EPS Synthesis, Biofilm Formation, and Plant Growth‐Promoting Abilities of the Endophytes *Pseudomonas* PD9R and *Rahnella Laticis* PD12R.” Environmental Science & Technology 57: 8728–8738. 10.1021/acs.est.2c08586. 37256822

[pce70631-bib-0039] Verbon, E. H. , and L. M. Liberman . 2016. “Beneficial Microbes Affect Endogenous Mechanisms Controlling Root Development.” Trends in Plant Science 21: 218–229. 10.1016/j.tplants.2016.01.013. PMC4772406 26875056

[pce70631-bib-0040] Vullaganti, N. , B. G. Ram , and X. Sun . 2025. “Precision Agriculture Technologies for Soil Site‐Specific Nutrient Management: A Comprehensive Review.” Artificial Intelligence in Agriculture 15: 147–161. 10.1016/j.aiia.2025.02.001.

[pce70631-bib-0041] Walker, V. , C. Bertrand , F. Bellvert , Y. Moënne‐Loccoz , R. Bally , and G. Comte . 2010. “Host Plant Secondary Metabolite Profiling Shows a Complex, Strain‐Dependent Response of Maize to Plant Growth‐Promoting Rhizobacteria of the Genus *Azospirillum* .” New Phytologist 189: 494–506. 10.1111/j.1469-8137.2010.03484.x. 20946131

[pce70631-bib-0042] Wang, L. , C. Ju , C. Han , Z. Yu , M. Y. Bai , and C. Wang . 2025a. “The Interaction of Nutrient Uptake With Biotic and Abiotic Stresses in Plants.” Journal of Integrative Plant Biology 67: 455–487. 10.1111/jipb.13827. 39783785

[pce70631-bib-0043] Wang, W. , T. Tian , M.‐Y. Li , et al. 2024a. “Carbon and Nitrogen Stoichiometry Across Plant–Soil System Accounts for the Degradation of Multi‐Year Alfalfa Grassland.” Frontiers in Plant Science 15: 1400261. 10.3389/fpls.2024.1400261. PMC11527612 39494059

[pce70631-bib-0044] Wang, W. , Y. Xia , P. Zhang , et al. 2025b. “Narrow‐Spectrum Resource‐Utilizing Bacteria Drive the Stability of Synthetic Communities Through Enhancing Metabolic Interactions.” Nature Communications 16: 6088. 10.1038/s41467-025-61432-7. PMC12222865 40603291

[pce70631-bib-0045] Wang, Y. , W. Li , H. Wang , and G. Lv . 2025c. “Rhizosphere Effect Under Drought Stress: To Explore Its Influence on Microbial Regulation of the Soil Carbon Cycle.” Applied Soil Ecology 216: 106543. 10.1016/j.apsoil.2025.106543.

[pce70631-bib-0046] Wang, Y. , Y. Yang , D. Zhao , et al. 2024b. “ *Ensifer* sp. GMS14 Enhances Soybean Salt Tolerance for Potential Application in Saline Soil Reclamation.” Journal of Environmental Management 349: 119488. 10.1016/j.jenvman.2023.119488. 37939476

[pce70631-bib-0047] Wei, K. , Y. Sun , A. D. Cartmill , I. F. López , C. Ma , and Q. Zhang . 2025. “Long‐Term Effects of Nitrogen and Phosphorus Fertilizers on Rhizosphere Physicochemical Characteristics and Microbial Composition in Alfalfa.” Industrial Crops and Products 227: 120776. 10.1016/j.indcrop.2025.120776.

[pce70631-bib-0048] Wen, T. , J. Yuan , X. He , Y. Lin , Q. Huang , and Q. Shen . 2020. “Enrichment of Beneficial Cucumber Rhizosphere Microbes Mediated by Organic Acid Secretion.” Horticulture Research 7: 154. 10.1038/s41438-020-00380-3. PMC7527982 33082961

[pce70631-bib-0049] Xu, F. , Y. Wang , J. Yang , et al. 2025. “Auxin‐Producing *Pseudomonas* Recruited by Root Flavonoids Increases Rice Rhizosheath Formation Through the Bacterial Histidine Kinase Under Soil Drying.” Advanced Science 12: e00607. 10.1002/advs.202500607. PMC12442626 40536439

[pce70631-bib-0050] Xu, Y. , X. Duan , Y. Wu , et al. 2024. “Carbon Sequestration Potential and Its Main Drivers in Soils under Alfalfa (*Medicago Sativa* L.” Science of the Total Environment 935: 173338. 10.1016/j.scitotenv.2024.173338. 38763193

[pce70631-bib-0051] Xueping, Y. , D. Dan , Z. Mengzhen , et al. 2025. “Pathogenic Fungi Accumulation Rather Than Direct Autotoxicity Is Responsible for the Root‐Rot Disease of Foxtail Millet Under Continuous Monoculture Regimes.” Biology and Fertility of Soils 61: 877–892. 10.1007/s00374-025-01904-6.

[pce70631-bib-0052] Yang, X. , R. Yuan , S. Yang , et al. 2024. “A Salt‐Tolerant Growth‐Promoting Phyllosphere Microbial Combination From Mangrove Plants and Its Mechanism for Promoting Salt Tolerance in Rice.” Microbiome 12: 270. 10.1186/s40168-024-01969-9. PMC11662529 39707568

[pce70631-bib-0053] Zhalnina, K. , K. B. Louie , Z. Hao , et al. 2018. “Dynamic Root Exudate Chemistry and Microbial Substrate Preferences Drive Patterns in Rhizosphere Microbial Community Assembly.” Nature Microbiology 3: 470–480. 10.1038/s41564-018-0129-3. 29556109

[pce70631-bib-0054] Zhang, F. , Y. Hou , R. Zed , et al. 2023. “Root Exudation of Organic Acid Anions and Recruitment of Beneficial Actinobacteria Facilitate Phosphorus Uptake by Maize in Compacted Silt Loam Soil.” Soil Biology and Biochemistry 184: 109074. 10.1016/j.soilbio.2023.109074.

[pce70631-bib-0055] Zhang, Y. , S. Y. Zhao , R. H. Zhang , et al. 2024. “Screening of Plant Growth‐Promoting Rhizobacteria Helps Alleviate the Joint Toxicity of PVC+Cd Pollution in Sorghum Plants.” Environmental Pollution 355: 124201. 10.1016/j.envpol.2024.124201. 38810675

[pce70631-bib-0056] Zhou, X. , J. Zhang , M. Khashi u Rahman , et al. 2023. “Interspecific Plant Interaction via Root Exudates Structures the Disease Suppressiveness of Rhizosphere Microbiomes.” Molecular Plant 16: 849–864. 10.1016/j.molp.2023.03.009. 36935607

[pce70631-bib-0057] Zhu, Y. , L. Wang , J. Ma , Y. Li , F. Chen , and W. Peijnenburg . 2023. “Comparative Physiological and Metabolomics Analyses Using Ag–NPs and HAS31 (PGPR) to Alleviate Cr Stress in Barley (*Hordeum Vulgare* L.” Environmental Pollution 333: 122010. 10.1016/j.envpol.2023.122010. 37302784

[pce70631-bib-0058] Ziegler, J. , S. Schmidt , R. Chutia , et al. 2015. “Non‐Targeted Profiling of Semi‐Polar Metabolites in Arabidopsis Root Exudates Uncovers a Role for Coumarin Secretion and Lignification During the Local Response to Phosphate Limitation.” Journal of Experimental Botany 67: 1421–1432. 10.1093/jxb/erv539. PMC4762384 26685189

